# Neurological Development, Epilepsy, and the Pharmacotherapy Approach in Children with Congenital Zika Syndrome: Results from a Two-Year Follow-up Study

**DOI:** 10.3390/v12101083

**Published:** 2020-09-25

**Authors:** Maria Eulina Quilião, Fabio Antonio Venancio, Lisany Krug Mareto, Sahra de Almeida Metzker, Ana Isabel do Nascimento, Daniele Cristina Vitorelli-Venancio, Cláudia Du Bocage Santos-Pinto, Everton Falcão de Oliveira

**Affiliations:** 1 Centro Especializado em Reabilitação, Associação de Pais e Amigos dos Excepcionais, Campo Grande 79050-140, Brazil; eulinab@hotmail.com; 2 Programa de Pós-Graduação em Doenças Infecciosas e Parasitárias, Universidade Federal de Mato Grosso do Sul, Campo Grande 79070-900, Brazil; venanciofab@gmail.com (F.A.V.); danielevitorelli@gmail.com (D.C.V.-V.); 3 Instituto Integrado de Saúde, Universidade Federal de Mato Grosso do Sul, Campo Grande 79070-900, Brazil; lisanykrugmareto@gmail.com (L.K.M.); bocage.santos@ufms.br (C.D.B.S.-P.); 4 Faculdade de Medicina, Universidade Federal de Mato Grosso do Sul, Campo Grande 79070-900, Brazil; sahramedicina@gmail.com; 5 Faculdade de Ciências Farmacêuticas, Alimentos e Nutrição, Universidade Federal de Mato Grosso do Sul, Campo Grande 79070-900, Brazil; ana.bel.nasc@gmail.com

**Keywords:** Zika virus, congenital Zika syndrome, epilepsy, West syndrome, motor impairment

## Abstract

Clinical outcomes related to congenital Zika syndrome (CZS) include microcephaly accompanied by specific brain injuries. Among several CZS outcomes that have been described, epilepsy and motor impairments are present in most cases. Pharmacological treatment for seizures resulting from epilepsy is performed with anticonvulsant drugs, which in the long term are related to impairments in the child’s neuropsychomotor development. Here, we describe the results from a two-year follow-up of a cohort of children diagnosed with CZS related to the growth of the head circumference and some neurological and motor outcomes, including the pharmacological approach, and its results in the treatment of epileptic seizures. This paper is part of a prospective cohort study carried out in the state of Mato Grosso Sul, Brazil, based on a Zika virus (ZIKV)-exposed child population. Our data were focused on the assessment of head circumference growth and some neurological and motor findings, including the description of seizure conditions and pharmacological management in two periods. Among the 11 children evaluated, 8 had severe microcephaly associated with motor impairment and/or epilepsy. Seven children were diagnosed with epilepsy. Of these, 3 had West syndrome. In four children with other forms of epilepsy, there was no pharmacological control.

## 1. Introduction

Zika virus (ZIKV) infection can result in unexpected neurological diseases and congenital malformations, with severe consequences for children exposed to prenatal ZIKV infection [1] and other outcomes in adults ranging from temporary mental confusion and motor disabilities to more severe outcomes, such as coma or memory loss [2].

Studies carried out between 2017 and 2019 indicate that there are approximately 5000 cases of children with congenital Zika syndrome (CZS) worldwide, distributed in 31 countries or territories [3,4]. In Brazil, the country with the highest number of cases, 3496 cases of CZS were confirmed in live-born babies, and 2665 children remain under investigation as of May 2020 [5].

The term CZS was adopted to describe a series of alterations in fetuses and live-born babies exposed to ZIKV during pregnancy. The clinical findings described initially included microcephaly accompanied by specific brain injuries, with subcortical calcifications [6,7] due to cell apoptosis observed during ZIKV replication in cells of the central nervous system of the developing fetus [8,9,10]. Later, other outcomes were observed and added to the definition of CZS, such as severe microcephaly with partially collapsed skull [11,12], ocular alterations [13,14] and congenital contractures [15], hydrocephalus [16], craniofacial disproportion [17], muscle tone, postural alterations, marked early hypertonia and symptoms of extrapyramidal involvement [13,18,19], pseudobulbar syndrome [20], and postnatal microcephaly [20,21,22].

Motor impairment, such as paresis, hyperreflexia, and hypertonia with spasticity, affects 77.3% to 100% of children with CZS [18,23,24,25]. Another abnormality frequently described is epileptic seizures, reported in 42% to 67% of children exposed to ZIKV and diagnosed with CZS [18,23,26,27]. Some authors suggest that the occurrence of these crises increases the probability of death in infants and children of preschool age [28,29]. Pharmacological treatment for seizures resulting from epilepsy is performed with anticonvulsant drugs, which in the long term are related to impairments in the child’s neuropsychomotor development [28].

This context reinforces the importance that individuals exposed to ZIKV are submitted to long-term clinical surveillance and highlights the urgent need for the development of therapeutic approaches to reduce or eliminate the neurological burden of infection [30]. Our study aimed to describe the results from a two-year follow-up of a cohort of children diagnosed with CZS related to the growth of head circumference and some neurological and motor outcomes. In addition, we describe the pharmacological approach and its results in the treatment of epileptic seizures.

## 2. Materials and Methods

### 2.1. Study Design and Participants

This paper is part of a prospective cohort study based on a ZIKV-exposed child population that we are carrying out in the state of Mato Grosso Sul, Brazil.

In the present study, the subjects were all children diagnosed as confirmed or potential cases of CZS in Mato Grosso do Sul between 2015 and 2018. The detailed methodology that led to the identification of these infants as well as the outcomes of the first year of the clinical assessment is described by Venancio et al. [20].

These children have been followed since October 2018 to assess their development, according to an established protocol focused on both cognitive and motor development, as reported by Venancio et al. [20]. For the present study, we focused on the following: (i) The growth of the head circumference; (ii) neurological and motor outcomes related to epilepsy, seizure, and gross motor function; and (iii) the description of the pharmacological approach for crisis control.

### 2.2. Procedures

At this time, two clinical assessments were carried out: The first between October 2018 and February 2019 and the second between October 2019 and February 2020. Data from these two time points and data at delivery were used to compare and characterize the follow-up.

For data on the evolution of head circumference and accurate follow-up results, we calculated the SD and Z-score in all children at delivery and in the clinical assessments performed by our research group, meeting the World Health Organization metrics [31] and the governmental criteria of the three measured periods [32].

A cerebral palsy diagnosis was established on a clinical basis, according to the definition and classification of cerebral palsy in April 2006 [33].

Seizures were classified according to the guidelines of the International League against Epilepsy [34]. For the description of seizures and pharmacological crisis control, we assessed the electroencephalogram (EEG) traces and reports present in the medical records, technical reports from other specialties, and family reports or videos of the patients who documented seizures.

Children with cerebral palsy were classified regarding gross motor skills using the Gross Motor Function Classification System (GMFCS) [35]. This five-level system was incorporated into our assessment protocol only in October 2019 (at the time of the second clinical assessment). Therefore, there are no previous data on this topic to be presented in this paper.

Descriptive analyses were conducted to identify the number and percent of infants according to the variables presented in this study. For categorical variables, absolute and relative frequencies were used. For continuous variables, we used the mean and standard deviation (SD). Relative change was used to evaluate head circumference and Z-score data from the two clinical assessments.

This study was approved by the Research Ethics Committee of the Federal University of Mato Grosso do Sul (CAAE: 91326518.1.0000.0021) and was registered under number 3.298.330. All individuals included and clinically assessed in this study signed the participant consent form. For the children, parents, or guardians of the participating children signed consent forms on the same day that the participants entered the study.

## 3. Results

A total of 11 cases of CZS were followed-up and assessed from October 2018 to February 2020. One child not reported in the first stage of the assessment was included in the paper (case 11). This is a premature live-born child in 2016, not reported in the RESP-Microcephaly (Brazilian Public Health Event Records for congenital anomalies due to STORCH and Zika) but with imaging tests suggestive of CZS. The child was born to a woman diagnosed with ZIKV infection during pregnancy, with laboratory confirmation by reverse transcriptase polymerase chain reaction (RT-PCR) and other teratogenic causes excluded.

At the time of the first assessment, the mean age of the children was 24 months (median = 27; SD = 7.57), and at the time of the second assessment, the mean age of the cohort was 36 months (median = 40; SD = 8.35).

Regarding the growth of the head circumference, 8 children showed a more severe microcephaly pattern at the last evaluation, with head circumference less than 4 or equal to 4 SD below the average for age (Table 1). This evidenced slower head growth between the period of birth (Z-score −4.53 to −1.12) and the second year of life (Z-score −8.84 to −4.8). Among these, 6 children showed a marked slowdown in head growth that persisted until the third assessment in 3 of these children (3, 4, and 5), who maintained a Z-score below 6 (Z-score −7.64 to −6.76). The child (case 8) described by Venancio et al. [20] with postnatal microcephaly maintained the head growth pattern between 2 years and 3 years of age with a Z-score of −4.80 and −4.73.

Data on EEG are shown in Table 2. Electroencephalographic tracing with a hypsarrhythmia pattern associated with epileptic spasms, compatible with West syndrome, stood out in 3 children.

In the first clinical assessment, 4 children had no history of seizures. After 1 year, they remained with no records of seizures. The other 7 children had been diagnosed with epilepsy at the first assessment, and 2 of them had changed their status and remained under investigation after the second assessment (cases 4 and 5) due to the absence of a seizure crisis for more than 25 months without the use of antiepileptic drugs (Table 2). The 5 children who still had convulsive activity all underwent pharmacological treatment, and in some cases, there were adjustments or drug changes made by the neuropediatrician who monitored them in the specialized outpatient clinic. However, only one (case 2) of these 5 children had adequate crisis control in this second year of assessment. The children (cases 7, 8, and 9) currently have greater complexity in their control of crises, with episodes of daily seizures, even with the change in medication and the adjustment of doses. Exceptionally, case 9 presented daily seizures up to the sixth month of life that were controlled with the use of oxcarbazepine, allowing up to 3 years of life without seizures. After 3 years of age, he started to present a daily frequency of seizures.

Based on the GMFCS scale measured at the second assessment, 9 children showed a level of 5, which indicates the highest degree of dependence and/or disability of gross motor functions. Cases 6 and 11 showed minor gross motor impairment with GMFCS levels of 3 and 2, respectively.

Table 3 summarizes and integrates the main findings of our report. Descriptively, it is observed that children with less motor impairment (cases 6 and 11) are those who presented the higher Z-score relative change, lower head circumference relative change, normal EEG tracing, and are seizure-free. For the others, it was not possible to observe a pattern of homogeneity or similarity among the variables evaluated in this study.

## 4. Discussion

Congenital Zika syndrome remains a public health problem, and due to the current age of these children who were exposed to ZIKV during the intrauterine period, the late outcomes are still not fully characterized [4,36]. Here, we describe the results of the second year of a longitudinal follow-up of a cohort of children with CZS. Our data were focused on the assessment of head circumference growth and some neurological and motor findings, including the description of seizure conditions and pharmacological management in two periods.

Our results showed that 8 children maintained the most severe degree of microcephaly, presenting 4 SD below the mean for age in the third measurement when the mean age of the cohort was 3 years. At this age, the growth of the head circumference is marked by two major growth peaks: The first occurs from the twelfth to the eighteenth week of gestation, and the second, and more importantly, occurs from the twenty-eighth week of gestation and extends to the third year of life [37]. Based on this, structural compensation for cranial growth was expected in this period, which did not occur.

A prospective study carried out with ZIKV-exposed children during pregnancy described the spontaneous resolution of microcephaly in a child in the second year of life [22]. In our cohort, conversely, we observed that in the period between birth and the second year of life, most children showed greater retardation in the growth of the head circumference, showing a much lower Z-score (Z-score −8.84 to −4.80) in relation to that measured at birth (Z-score −4.53 to −1.12). In a study carried out with primates, Raper et al. [38] reported that until the sixth month after the experimental postnatal infection, damage processes in the developing brain were still observed, even with the virus undetectable in the blood serum 7 days after the infection. This demonstrates that ZIKV infection causes a progressive damage process in the brain that can persist for months, causing structural and functional anomalies.

The replication of ZIKV in brain tissues after the birth of infected children during the intrauterine period [39,40] also points to possible long-term late actions resulting from infection. Based on the assumption that cranial growth is intrinsically related to adequate brain growth [41,42,43], we hypothesize that intrauterine changes related to ZIKV infection would manifest at later postnatal stages, mainly during the first months of life, when the greatest peak of cranial growth is expected. It is possible that this is related to the long-lasting effects resulting from death and/or damage to neural stem cells in utero that impacts postnatal neurogenesis and also the possibility of the persistence of viral replication in brain tissues. This event would explain the negative Z-score of the more marked head circumference in relation to the measurement at birth, as demonstrated in 8 children. In this sense, this hypothesis would be in line with cases of deceleration in the head circumference and cases of postnatal microcephaly, as demonstrated in one of the children in this cohort (case 7) in a previous report from our group [20] and by other authors [21,22,44].

Epilepsy has been described in several cohorts of children with CZS [20,23,24,45,46], and the frequency of seizures is quite variable, with rates ranging from 9% to 67% [23,26,45,46], in accordance with our data.

Children with no seizure control were also those with the most severe degree of microcephaly among the cohort. In addition, children with no seizure history or successful seizure control were those with less severe microcephaly among the cohort (except for child 1).

Generalized focal seizures and epileptic spasms are the types of seizures most frequently described [45]. West syndrome has been linked to the most severe cases of epilepsy in these children [26]. Epilepsy of infectious aetiology is the most commonly found worldwide, and some may have a structural correlation, as in the case of epilepsies resulting from congenital infections by cytomegalovirus and ZIKV [47,48].

Our second assessment showed that two children described with epilepsy by Venancio et al. [20] had their seizure activity controlled, and after having been off drugs for more than 15 months, no epileptic seizures were reported since then. The probability of the recurrence of crises is described in 60% of cases in children in general [49]. The other five children in our cohort remained on pharmacological treatment for epilepsy; however, only one child, who used vigabatrin, had a good pharmacological response showing seizure control. This was a focal epilepsy case, corroborating other studies that showed a better response to treatment in this type of epilepsy and the difficulty in controlling epileptic encephalopathy with spasm [50].

The other 4 children proved to be refractory to the proposed pharmacological treatments. Three of them present an EEG with a hypsarrhythmia activity pattern. Carvalho et al. [50] also observed a low rate of response to treatment (46.1%), and epileptic encephalopathy with spasm cases was shown to be refractory to several drug combinations.

The findings observed in the EEG and in the seizure outcome for the case 10 could indicate the presence of a lesion due to ZIKV infection, but this injury probably did not occur in an epileptogenic area. This fact may explain the non-occurrence of seizures. Descriptions of EEG findings in children with CZS have been limited, but one case series and another cohort study describe that 29% and 11% of children with CZS have a hypsarrhythmia pattern [26,46]. Although hypsarrhythmia found in the EEG in CZS is similar to the patterns most often described in malformations of cortical development caused by other aetiologies, in CZS, they appear to be more complex and may reflect continuous lesions [51].

The pattern of hypsarrhythmia found in these children points to West syndrome, which is characterized by the triad that includes seizures of spasm, delayed neuropsychomotor development, and the hypsarrhythmia pattern in the EEG [52]. West syndrome is an age-dependent epileptic encephalopathy and a multi-etiological condition, with diverse genetic, teratogenic, perinatal, and postnatally acquired etiological factors, such as cortical dysplasias, infections, asphyxia, or metabolic and chromosomal anomalies [53]. It has an incidence of 2 to 4.5/10,000 live births [54,55], and cognitive deterioration is present in 95% of cases, which is related to great losses in neuropsychomotor development. Treatment is still widely discussed in the medical literature, and there is no consensus on drugs of the first choice [52,56,57].

Lack of control of epileptic seizures can cause cognitive, motor, psychological, and social damage to patients [58]. Development, with constant and intense uncontrolled epileptic conditions, is currently the greatest cause for concern, anguish, and fear in the families of children with CZS.

In our cohort, most of the children were aged over 3 years at the time of the second assessment described in this paper and, in all, profound delays were found in all domains of neuropsychomotor development, as has also been demonstrated in several studies with children exposed to ZIKV [15,22,28,59]. Only two children described did not have microcephaly, but they did have severe neuropsychomotor delays. It reinforces the multiple CZS manifestations forms and consequences that, in some cases, can only be identified in later phases of child development [15,21].

In several studies that have focused on the description of abnormal neurological and motor development in ZIKV-exposed children during pregnancy, serious motor damage has been described [22,59]. In our cohort, the GMFCS scale showed a more severe level (level 5) in microcephalic children, pointing to motor impairment and the inability to perform basic functions, such as sitting, standing, and even maintaining antigravity postures of the head and trunk, as demonstrated in previous studies [28].

Motor disabilities are clearly linked to the extent of damage to the central nervous system [15,28]. A retrospective review on imaging tests (computed tomography and magnetic resonance) of children with CZS under 1 year of age described that brain damage differs among children with microcephaly at birth, those with postnatal microcephaly, and those without microcephaly [21]. These findings also support our results from the GMFCS scale concerning children without microcephaly who had a better result, varying between grades 2 and 3 on the GMFCS scale, in performing some functions not performed by children in the microcephalus group. Descriptively, we did not identify any motor differences between children with prenatal-onset and postnatal microcephaly.

Here, we provide important data from a cohort of children with CZS, such as severe retardation in head growth between the period of birth and the second and third years of life. We report through gross motor assessment that 75% of children have the highest level of motor disability. We also demonstrated that 58% of children in this cohort have an epileptic outcome. In general, we describe clinical findings that assist in clinical and medical practice and that, in a way, contribute to clarifying the mechanism of the in-progress disease and its outcomes.

As this is a longitudinal study, it was possible to describe the assessment of the outcomes and even to compare their evolution in different periods. However, it is necessary to carry out studies that include a control group in the longitudinal follow-up to compare the findings and estimate the risk of negative outcomes in children.

## Figures and Tables

**Table 1:** Longitudinal monitoring of head circumference of children with congenital Zika syndrome (CZS).

ID		At Delivery			First Assessment (Oct 2018 to Feb 2019)			Second Assessment (Oct 2019 to Feb 2020)		
	CZS Status	Gestation Length (in Week)	Head Circumference	SD (Z-Score)	Child’s Age	Head Circumference	SD (Z-Score)	Child’s Age	Head Circumference	SD (Z-Score)
1	Confirmed	39	29	−3 (−3.89)	2y 3m	42	−4 (−4.80)	3y 3m	43.5	−4 (−4.32)
2	Confirmed	42	31	−3 (−3.40)	2y 3m	39	−4 (−6.98)	3y 4m	44	−4 (−4.00)
3	Confirmed	39	28	−4 (−4.29)	2y 3m	38	−4 (−6.84)	3y 3m	38	−4 (−7.60)
4	Confirmed	35	28	−3 (−3.16)	2y 4m	36.5	−4 (−8.84)	3y 3m	40	−4 (−6.76)
5	Confirmed	36	26	−4 (−4.53)	2y 4m	36	−4 (−8.34)	3y 4m	38	−4 (−7.64)
6	Potential	41	35.5	0 (+0.70)	2y 4m	49	0 (+0.20)	3y 7m	50	0 (+0.04)
7	Potential	34	26	−4 (−3.79)	0y 7m	36	−4 (−5.19)	1y 10m	39	−4 (−5.67)
8	Potential	37	32	−1 (−1.12)	2y 3m	42	−4 (−4.80)	3y 5m	43	−4 (−4.73)
9	Potential	39	30	−3 (−3.22)	2y 3m	42	−4 (−4.80)	3y 6m	43.5	−4 (−4.42)
10	Potential	35	29	−3 (−2.15)	0y 9m	40	−2 (−2.86)	1y 5m	43	−2 (−2.22)
11	Potential	24	29	+4 (+4.10)	2y 5m	48	0 (+0.13)	3y 7m	49	0 (−0.02)

ID: Identification number; SD = Standard deviation; Confirmed case: A live-born child with clinical outcomes and imaging evidence suggestive of CZS, RT-PCR ZIKV-positive, or ZIKV-reagent serology (IgM) tested after birth and with unreacted/negative STORCH results in both the mother and newborn; Potential case: A live-born child with clinical outcomes and imaging evidence suggestive of CZS, with ZIKV-reagent serology (IgG) after birth, and/or live-born from a ZIKV-positive mother (RT-PCR or ZIKV-reagent serology—IgM), who has clinical outcomes and suggestive imaging evidence of CZS.

**Table 2:** Electroencephalogram, occurrence of epilepsy, type, treatment, and seizure control of congenital Zika syndrome cases.

ID	EEG	First Assessment (Oct 2018 to Feb 2019)					Second Assessment (Oct 2019 to Feb 2020)				
		Child’s Age (First Evaluation)	Seizure Frequency	Seizure Type	Treatment	Seizure Control	Child’s Age (Second Evaluation)	Seizure Frequency	Seizure Type	Treatment	Seizure Control
1	Normal	2y 3m	No seizure	No epileptic	No treatment	No applicable	3y 3m	No seizure	No epileptic	No treatment	No applicable
2	Focal	2y 3m	Weekly seizures	Focal motor	Monotherapy (phenobarbital)	No	3y 4m	No seizure	Controlled epilepsy	Monotherapy (vigabatrin)	Yes
3	Hypsarrhythmia	2y 3m	Daily seizures	Epileptic spasms	Polytherapy (sodium valproate, vigabatrin, and phenobarbital)	No	3y 3m	Occasionally	Focal motor	Polytherapy (levetiracetam and phenobarbital)	No
4	Generalized epileptiform discharges	2y 4m	Single seizure (at 14 months)	Tonic-clonic	Monotherapy (phenobarbital)	Yes	3y 3m	No seizure	No seizure	No treatment	No applicable
5	Focal	2y 4m	Single seizure (at 4 months)	Focal motor	Monotherapy (phenobarbital)	Yes	3y 4m	No seizure	No seizure	No treatment	No applicable
6	Normal	2y 4m	No seizure	No epileptic	No treatment	No applicable	3y 7m	No seizure	No epileptic	No treatment	No applicable
7	Hypsarrhythmia	0y 7m	Daily seizures	Epileptic spasms	Polytherapy (clonazepam, levetiracetam, and vigabatrin)	No	1y 7m	Daily seizures	Epileptic spasms	Polytherapy (vigabatrin and nitrazepam)	No
8	Hypsarrhythmia	2y 3m	Daily seizures	Epileptic spasms	Polytherapy (levetiracetam, vigabatrin, and phenobarbital)	No	3y 5m	Daily seizures	Epileptic spasms	Polytherapy (levetiracetam, vigabatrin, and clobazam)	No
9	Multifocal epileptiform discharge	2y 3m	Weekly seizures up to 6 months old	Focal motor	Monotherapy (oxcarbazepine)	Yes	3y 6m	Daily seizures starting at 3 years old	Epileptic spasms	Polytherapy (oxcarbazepine and levetiracetam)	No
10	Focal	0y 9m	No seizure	No epileptic	No treatment	No applicable	1y 5m	No seizure	No epileptic	No treatment	No applicable
11	Normal	2y 5m	No seizure	No epileptic	No treatment	No applicable	3y 7m	No seizure	No epileptic	No treatment	No applicable

ID: Identification number; EEG: Electroencephalogram.

**Table 3:** Head circumference and Z-score relative changes and other main study findings.

ID	HC Relative Change (%)	Z-Score Relative Change (%) *	Microcephaly	EEG	Seizure Occurrence	Seizure Control after the Two Assessments	GMFCS
1	3.6	10.0	Yes	Normal	No	No applicable	5
2	12.8	42.7	Yes	Focal	Yes	Yes	5
3	0.0	11.1	Yes	Hypsarrhythmia	Yes	No	5
4	9.6	23.5	Yes	Generalized epileptiform discharges	Yes	Yes	5
5	5.6	8.4		Focal	Yes	Yes	5
6	2.0	80.0	No	Normal	No	No applicable	3
7	8.3	9.2	Yes	Hypsarrhythmia	Yes	No	5
8	2.4	1.5	Yes	Hypsarrhythmia	Yes	No	5
9	3.6	7.9	Yes	Multifocal epileptiform discharge	Yes	No	5
10	7.5	22.4	No	Focal	No	No applicable	5
11	2.1	115.4	No	Normal	No	No applicable	2

* The relative difference was expressed in module values. ID: Identification number; EEG: Electroencephalogram; GMFCS: Gross Motor Function Classification System.
